# Direct-acting antivirals for acute hepatitis C in HIV-infected MSM

**DOI:** 10.1097/QAD.0000000000001157

**Published:** 2016-07-26

**Authors:** James Daniel Millard, Jaimie Henry, Syed Shoaib Rizvi, Mark Nelson

**Affiliations:** a Chelsea and Westminster Hospital, London; b Wellcome Trust Liverpool Glasgow Centre for Global Health Research, University of Liverpool, Liverpool; c Faculty of Medicine, Imperial College London, South Kensington Campus, London, UK.

An epidemic of acute hepatitis C (AHC) has been described amongst HIV-infected MSM [1–8]. Traditionally, treating AHC in this population has had clear advantages over waiting to treat in the chronic phase, with improved sustained virological response (SVR) rates and reduced length of therapy [9–12]. Treatment is offered to those who fail to demonstrate a 2 log decline at week 4 or still have detectable hepatitis C virus (HCV) RNA at week 12 and hence are unlikely to clear spontaneously [13]. The treatment of chronic HCV has been revolutionized by the advent of directly acting antivirals (DAAs) [14]. However, the role of these agents in AHC is unclear, particularly in light of the now-excellent efficacy in chronic infection. Guidelines still recommend therapy with pegylated interferon and ribavirin, and DAAs are not currently licensed for AHC [9,15]. Nonetheless, we have encountered several cases in which we have felt the use of DAAs warranted for AHC. The demographics and essentials of the HIV and AHC history of these patients are presented in Table 1.

Patient 1 was monitored for 4 weeks, failed to reduce his viral load by 2 log and was thus considered for AHC therapy. He had a background of depression and was a healthcare professional in an important role. Two companies were therefore approached to access an all-oral, interferon-free DAA regimen, as DAAs were not licensed at this time, both of whom agreed to provide medication. Patient 2 presented with evidence of hepatic failure, with deranged clotting and low albumin, which failed to resolve after more than 10 days of supportive inpatient therapy. Similarly, although patient 4 had initially normal synthetic function, after a week he developed hepatic failure. Given the clinical severity and, in the case of patient 2, background of depression, we aimed to avoid interferon-based therapy and obtained access to DAAs. Patient 3 was concerned about transmission risk, keen to initiate therapy and had access to DAAs privately.

All patients were commenced on Harvoni, a fixed dose combination of 90 mg of the NS5A inhibitor ledipasvir and 400 mg of the nucleotide analogue NS5B polymerase inhibitor sofosbuvir, once per day. Length of therapy and the addition (or otherwise) of ribavirin was based on current understanding of the length of therapy required for AHC at the time of treatment initiation, HCV RNA viral load and evidence of underlying liver disease; with patient 1 (low viral load, no underlying liver disease and prior to evidence for the possible efficacy of shorter courses [16]) receiving 12 weeks of Harvoni alone, patients 2 and 4 (high viral load and evidence of underlying liver disease on the basis of Fibroscan or liver biopsy) commencing 12 weeks of Harvoni and ribavirin and patient 3 (modest viral load, no underlying liver disease and after publication of evidence for the possible efficacy of shorter courses [16]) receiving only 8 weeks of Harvoni.

Three patients (1, 2 and 4) were receiving protease inhibitor therapy for their HIV infection at the time of the AHC diagnosis. To minimize any potential drug–drug interactions and reduce any liver toxicity, patients 1 and 2 had their HIV therapy switched to integrase inhibitor based therapy (Truvada and Raltegravir in the case of patient 1 and Triumeq, after a 3-week pause to allow liver function test recovery, in the case of patient 4). Patient 2 had extensive previous antiretroviral exposure and current hepatic failure, and hence antiretrovirals were discontinued until complete normalization of liver function tests and completion of DAA therapy, at which point, the same antiretrovirals were recommenced. Patient 3 was receiving an non-nucleoside reverse-transcriptase inhibitor-based regimen and had only a modest alanine transaminase (ALT) rise and hence remained on the same antiretrovirals throughout.

Aside from patient 2, who had to discontinue ribavirin after 2 weeks due to nausea, all patients had an uncomplicated follow-up, requiring only three to five short visits. Liver function tests and HCV RNA monitoring were variable and dictated by clinical need, all patients had no detectable HCV RNA and normal ALT by week 8, and those with lower baseline HCV RNA became undetectable sooner. Patients 1, 2 and 4 have achieved an SVR_12_, and patient 3 has achieved an SVR_4_.

The optimum regimen and duration of therapy for AHC are unclear, particularly in the light of recent, poorer than expected results, for both 6- and 12-week regimens of sofosbuvir and ribavirin [17,18]. However, our experience offers proof of principle for well tolerated, all-oral DAA therapy with Harvoni (with and without ribavirin) across a range of clinical situations and with minimal follow-up.

## Figures and Tables

**Table 1:** Demographics, HIV, acute hepatitis C history and therapy for four patients treated with directly acting antivirals for acute hepatitis C.

Demographics	HIV history	AHC history	Hepatitis C therapy
1: Man, 37 years, MSM	Diagnosed: unknown	Presentation: Incidental finding of raised ALT	12 weeks Harvoni (initiated ∼4 weeks postdiagnosis)
	Nadir CD4^+^ cell count: unknown	Initial blood results: ALT 295	HCV RNA:
	Current: CD4^+^ cell count 785, viral load <40	Genotype: 1a	Week 4: ND
	Resistance: nil	HCV RNA: 92 526 IU/ml (log^10^ 4.97)	Week 12: ND
	Current ARVs: Atazanavir/r, Truvada		SVR_12_
2: Man, 56 years, MSM	Diagnosed: 1988	Presentation: Jaundice and nausea	12 weeks Harvoni and ribavirin (initiated ∼2 weeks postdiagnosis, ribavirin discontinued after ∼2 weeks)
	Nadir CD4^+^ cell count: 25	Initial blood results: Bil 187, ALT 1894, ALP 165, Alb 28, PT 15.6 and APTT 36.4	HCV RNA:
	Current: CD4^+^ cell count 439 (24.1%), viral load <40	Fibroscan: >70 kPa	Week 1: 16 544 IU/ml
	Resistance: Nil	Transjugular liver biopsy: HVPG = 10 mmHg (normal <5 mmHg), focal bridging fibrosis, severe acute lobular hepatitis and no steatosis	Week 2: 2286 IU/ml
	Current ARVs: Atazanavir/r, Truvada	Genotype: 1a	Week 8: ND
		HCV RNA: 27 537 437 IU/ml (log^10^ 7.44)	SVR_12_
3: Man, 52 years, MSM	Diagnosed: 2011	Presentation: Investigation of raised ALT	8 weeks Harvoni (initiated 6 weeks postdiagnosis)
	Nadir CD4^+^ cell count: unknown	Initial blood results: Bil 12, ALT 276, 128 and Alb 36	HCV RNA:
	Current: CD4^+^ cell count 689 (37.1%), viral load <40	Genotype: 1a	Week 2: 89 IU/ml
	Resistance: Nil	HCV RNA: 2301 877 IU/ml (log^10^ 6.36)	SVR_4_
	Current ARVs: Eviplera		
4: Man, 42 years, MSM	Diagnosed: 1998	Presentation: Jaundice, pale stools, dark urine, nausea and fatigue	12 weeks Harvoni and ribavirin (initiated ∼10 days postdiagnosis)
	Nadir CD4^+^ cell count: 497 (38.1%)	Initial blood results: Bil 411, ALT 2431, ALP 110, Alb 37, PT 12.2 and APTT 27.4	HCV RNA:
	Current: CD4^+^ cell count 573 (32.8%), viral load <40	Fibroscan: >70 kPa, improved to 11.8 kPa at week 2	Week 1: 11 274 IU/ml
	Resistance: K103N	Genotype: 1a	Week 2: 1026 IU/ml
	Current ARVs: Darunavir/r	HCV RNA: 68 977 554 IU/ml (log^10^ 7.84)	Week 4: 163 IU/ml
			Week 8: ND
			Week 12: ND
			SVR_12_

AHC, acute hepatitis C; Alb, albumin; ALP, alkaline phosphatase; ALT, alanine transaminase; APTT, activated partial thromboplastin time; Bil, bilirubin; HVPG, hepatic venous pressure gradient; ND, not detected; PT, prothrombin time; Harvoni; ledipasvir 90 mg/sofosbuvir 400 mg; SVR, sustained virological response.
